# Baclofen decreases compulsive alcohol drinking in rats characterized by reduced levels of GAT‐3 in the central amygdala

**DOI:** 10.1111/adb.13011

**Published:** 2021-02-01

**Authors:** Lucia Marti‐Prats, Aude Belin‐Rauscent, Maxime Fouyssac, Mickaël Puaud, Paul J Cocker, Barry J Everitt, David Belin

**Affiliations:** ^1^ Department of Psychology University of Cambridge Cambridge UK

**Keywords:** alcohol, baclofen, central amygdala, compulsive alcohol drinking, GABA transporter GAT‐3

## Abstract

While most individuals with access to alcohol drink it recreationally, some vulnerable individuals eventually lose control over their intake and progressively develop compulsive alcohol drinking and decreased interest in alternative sources of reinforcement, two key features of addiction. The neural and molecular mechanisms underlying this vulnerability to switch from controlled to compulsive alcohol intake have not been fully elucidated. It has been shown that rats having reduced levels of expression of the gamma‐aminobutyric acid (GABA) transporter, GAT‐3, in the amygdala tend to persist in seeking and drinking alcohol even when adulterated with quinine, suggesting that pharmacological interventions aimed at restoring GABA homeostasis in these individuals may provide a targeted treatment to limit compulsive alcohol drinking. Here, we tested the hypothesis that the GABA_B_ receptor agonist baclofen, which decreases GABA release, specifically reduces compulsive alcohol drinking in vulnerable individuals. In a large cohort of Sprague–Dawley rats allowed to drink alcohol under an intermittent two‐bottle choice procedure, a cluster of individuals was identified that persisted in drinking alcohol despite adulteration with quinine or when an alternative ingestive reinforcer, saccharin, was available. In these rats, which were characterized by decreased GAT‐3 mRNA levels in the central amygdala, acute baclofen administration (1.5 mg/kg, intraperitoneal) resulted in a decrease in compulsive drinking. These results indicate that low GAT‐3 mRNA levels in the central amygdala may represent an endophenotype of vulnerability to develop a compulsive drinking of alcohol that is shown here to be mitigated by baclofen.

## Graphical abstract

In a large cohort of rats allowed to drink alcohol under an intermittent two‐bottle choice procedure, a cluster of individuals was identified that persisted in drinking alcohol despite adulteration or the availability of an alternative ingestive reinforcer, saccharin. In these rats, that were characterized by decreased GAT‐3 mRNA levels in the central amygdala, acute baclofen administration resulted in a decrease in compulsive alcohol drinking.

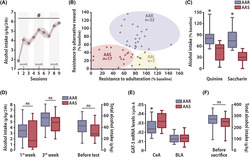

## 1 INTRODUCTION

About 2.3 billion people worldwide consume alcohol.^1^ While most maintain control over their recreational drinking, a subset of individuals is vulnerable to progressively develop compulsive alcohol seeking and drinking that persists despite adverse consequences and to the detriment of other activities and sources of reinforcement.^2^, ^3^, ^4^, ^5^ Given the significant health burden that alcohol addiction places on our society, estimated to cause 7.2% of the premature deaths worldwide,^1^ a better understanding of the neural, cellular, and molecular basis of the vulnerability to shift from recreational to compulsive alcohol drinking may enable novel treatment targets to be identified.

Many preclinical and clinical studies have highlighted the involvement of the gamma‐aminobutyric acid (GABA) system in the regulation of the reinforcing properties of alcohol and in the development of alcohol addiction. Pharmacological manipulations of GABAergic transmission through targeting both GABA_A_ and GABA_B_ receptors influence different behavioral responses to alcohol, including alcohol self‐administration, voluntary alcohol drinking, relapse‐like and binge‐like alcohol drinking, and the motivation for alcohol.^6^, ^7^, ^8^, ^9^, ^10^ Furthermore, alterations in the expression of genes encoding some GABA_A_ and GABA_B_ receptor subunits, as well as GABA transporters, have been shown to influence the predisposition to high alcohol consumption and vulnerability to alcohol use disorder (AUD) in humans, as well as AUD‐like behavior in rodents.^11^, ^12^

The central nucleus of the amygdala (CeA) is a key neural locus in which the GABAergic system mediates neuroadaptations involved in alcohol drinking behaviors.^13^, ^14^ Both acute and chronic alcohol exposure increase GABAergic transmission in the CeA and rats made dependent by chronic exposure to alcohol vapor show increased baseline GABA levels in the CeA compared to alcohol‐naïve rats.^13^, ^14^ Alcohol self‐administration is reduced by microinjection of a GABA_A_ receptor agonist into the CeA of rats with this history of exposure to alcohol vapor,^15^ whereas, by contrast, it is reduced by a GABA_A_ receptor antagonist^16^ in rats without. These data indicate that chronic exposure to alcohol is associated with adaptations in the GABAergic system within the CeA.

Beyond its involvement in controlling alcohol drinking, the GABAergic system within the CeA has also been shown to be involved in the development of addiction‐like behaviors for alcohol. Augier et al.^17^ recently revealed a downregulation of the mRNA level for several genes involved in GABA homeostasis in a subpopulation of rats that both preferred alcohol over an alternative reinforcer in an exclusive‐choice instrumental procedure and also showed the addiction‐like behaviors^18^, ^19^ of a high motivation for alcohol and the persistence of drinking when alcohol was adulterated with the bitter tastant, quinine.^17^ Downregulation of the GABA transporter GAT‐3 in the amygdala was also shown to be associated both with the choice of alcohol over an alternative ingestive reward and with compulsive alcohol drinking. GAT‐3 downregulation was also found post‐mortem in the CeA of brains of individuals having been diagnosed with AUD.^17^ Together, these data strongly suggest that alterations in the expression of GAT‐3 could be a relevant candidate in the search of markers of vulnerability to and also a treatment target for alcohol addiction.

Functionally, GABA transporters mediate GABA uptake and control extracellular GABA levels and GABAergic transmission in the central nervous system,^20^, ^21^ and inhibition of the GABA transporters results in an increase of extracellular levels of GABA.^20^ This is consistent with the finding that rats having a propensity to develop compulsive alcohol seeking and drinking that are characterized by decreased expression of GAT‐3 also show increased GABAergic tone in the CeA.^17^ This indicates that restoring the putatively altered extracellular GABA levels triggered by reduced GAT‐3 expression may decrease persistent alcohol drinking in compulsive individuals.^17^, ^22^ For example, GABA receptor agonists such as baclofen may restore extrasynaptic GABA levels by acting at presynaptic GABA_B_ receptors that control GABA release in the synaptic cleft^22^, ^23^, ^24^ and hence decrease alcohol drinking.

Activation of GABA_B_ receptors has been shown to prevent preparatory and consummatory responses for alcohol, such as the acquisition and maintenance of voluntary alcohol drinking, binge‐like alcohol drinking, and instrumental responding for alcohol.^6^, ^8^, ^9^, ^10^ Adaptations to chronic alcohol exposure such as the alcohol deprivation effect and the reinstatement of extinguished alcohol‐seeking behavior are also prevented by GABA_B_ receptor activation.^6^, ^8^ However, the influence of GABA_B_ receptor agonists on compulsive alcohol drinking, persisting in vulnerable individuals in the face of adulteration with quinine,^17^, ^25^, ^26^, ^27^, ^28^, ^29^, ^30^, ^31^ has not yet been investigated.

Here, we hypothesized that GABA_B_ agonists may rescue GAT‐3‐related deficits in GABA homeostasis associated with the vulnerability to develop compulsive alcohol drinking. We, therefore, investigated the effects of systemically administered baclofen on alcohol drinking in rats identified as resistant or sensitive to adulteration/alternative reward availability (i.e., compulsive or non‐compulsive, respectively) following a long history of exposure to alcohol under an intermittent two‐bottle choice procedure.

The results show that rats characterized by lower GAT‐3 mRNA levels in the CeA tended to persist in drinking alcohol despite the availability of an alternative ingestive sweet reward and adulteration with quinine, two behavioral features of compulsive alcohol drinking.^2^, ^3^, ^29^, ^32^, ^33^ We further show that while baclofen decreased voluntary alcohol intake in all rats, it selectively decreased the quinine‐resistant, compulsive, drinking of alcohol in vulnerable rats.

## 2 MATERIALS AND METHODS

### 2.1 Subjects

Forty‐eight adult male Sprague–Dawley rats (Charles River Laboratories, Kent, UK) weighing approximately 250 g on arrival were single‐housed in a temperature (22 ± 1°C) and humidity (60 ± 5%) controlled environment, under a 12‐h reverse light/dark cycle (light on at 7:00 p.m.) with ad libitum access to food (standard chow) and water. After 2 weeks of habituation to the vivarium and prior to being given access to alcohol, rats were progressively food restricted to reach 85%–90% of their free‐feeding body weight,^34^, ^35^ conditions previously shown not to influence alcohol intake.^36^

All procedures were conducted under the project license number PPL 70/8072, in accordance with the United Kingdom Animals (Scientific Procedures) Act 1986, amendment regulations 2012 following ethical review by the University of Cambridge Animal Welfare and Ethical Review Body (AWERB).

### 2.2 Experimental procedures

After two weeks of habituation to the animal facility followed by two weeks of food restriction, rats (*n* = 48) were given intermittent access to alcohol in a two‐bottle choice procedure.^37^, ^38^ One individual failed to acquire alcohol drinking and was therefore excluded from all analyses (*n* = 47). After three weeks of intermittent access to alcohol in the two‐bottle choice procedure,^37^, ^38^ the compulsive nature of alcohol drinking was assessed over successive challenge sessions during which the persistence of drinking alcohol was measured when alcohol was adulterated with quinine (session 10)^29^, ^31^, ^32^, ^39^ or in the presence of an alternative reward,^17^, ^30^, ^33^ saccharin (session 14). These two tests were separated by three baseline sessions. Previous studies have shown that exposure to saccharin prior to or after a long history of alcohol exposure does not influence the tendency to choose one over the other.^17^

**FIGURE 1:**
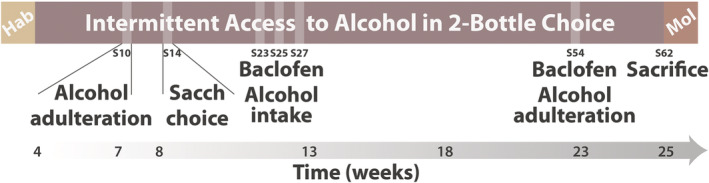
Timeline of the experiments. After 4 weeks of habituation to the vivarium, male Sprague–Dawley rats were given intermittent access to a 10% alcohol in a two‐bottle choice procedure. After 3 weeks of alcohol exposure, the compulsive nature of drinking behavior was assessed over successive challenge sessions during which the propensity of each individual to persist in drinking alcohol despite adulteration with quinine (session 10) or an alternative reward, saccharin (session 14), was measured. Three subpopulations, namely Adulteration‐Alternative reward Resistant (AAR), Intermediate and Sensitive (AAS), were characterized by K‐means clustering. Rats were re‐baselined in the intermittent two‐bottle choice procedure for 3 weeks, and the effect of baclofen (1 and 1.5 mg/kg) on voluntary alcohol drinking was measured for the entire population following a Latin square within‐subject design with a baseline session between treatments (baclofen/alcohol intake, sessions 23 to 27). Rats were then re‐baselined for 9 weeks, and the effect of baclofen on the persistence in alcohol drinking despite adulteration for the AAR and AAS subpopulations was assessed following a between‐subject design (baclofen/alcohol adulteration, session 54). Lastly, rats were re‐baselined before being sacrificed (session 62) and their brains harvested for posterior molecular analysis. Hab: habituation; Mol: molecular analysis; s: session; Sacch choice: saccharin choice

Adulteration‐Alternative reward Resistant, Intermediate, and Sensitive phenotypes (AAR, AAI and AAS, respectively) were characterized based on the persistence in drinking alcohol in both tests by a k‐mean cluster analysis.^40^, ^41^, ^42^ Subsequently, rats were re‐baselined in the intermittent two‐bottle choice procedure (alcohol vs. water) over three weeks and habituated to receive intraperitoneal (IP) administrations with saline. Then, the effect of baclofen on voluntary alcohol intake was measured over three sessions following a Latin square design with one baseline session between each administration (sessions 23 to 27). The population received IP injections of saline (0), baclofen 1 mg/kg (1) and 1.5 mg/kg (1.5) (*n* = 47/dose) 30 min prior to the beginning of the two‐bottle choice session.^10^ Thereafter, rats were re‐baselined for nine weeks prior to be subjected to a single alcohol adulteration test (session 54) during which the effect of baclofen on compulsive drinking was tested in a between‐subject design. For subsequent analyses, only the AAR and AAS subpopulations were considered. Each AAR and AAS subpopulation was split into two groups each, matched for their level of alcohol intake at baseline, which received IP administration of saline (AAR, *n* = 13; AAS, *n* = 8) or baclofen 1.5 mg/kg (AAR, *n* = 10; AAS, *n* = 9) 30 min before starting the two‐bottle choice session on which alcohol was adulterated with the bitter tastant quinine. Rats were re‐baselined for three  weeks (up to session 62) prior to being sacrificed and their brains harvested for the subsequent molecular analysis. GAT‐3 mRNA levels were measured both in CeA and basolateral amygdala (BLA) of rats from both AAS and AAR groups. One CeA and two BLA samples were not included in the final analysis due to technical issues, so that the final sample size was AAR: *n* = 22 and *n* = 21 versus AAS: *n* = 17 and *n* = 17 for CeA and BLA, respectively (Figure 1).

### 2.3 Drugs

A 10% (v/v) alcohol solution was prepared by mixing 99.8% ethanol (Sigma‐Aldrich) in tap water. Quinine hydrochloride dihydrate (Sigma‐Aldrich) was dissolved in 10% alcohol at the concentration of 0.1 g/L.^43^ Sodium saccharin (Sigma‐Aldrich) was dissolved in tap water at the concentration of 0.2% (v/v).^17^, ^44^

(±)‐Baclofen (Sigma‐Aldrich, UK) was freshly prepared, formulated at 1 and 1.5 mg/kg, dissolved in sterile 0.9% saline. Baclofen was administered IP in a volume of 1 ml/kg. These doses of baclofen have been shown in rats to yield no unspecific effects^9^, to result in plasma concentrations ~2,000 and 500 ng/mL after 30 and 180 min, respectively and to be linearly related to the ability of baclofen to decrease instrumental responding for alcohol.^45^

### 2.4 Intermittent access to two‐bottle choice for alcohol

Outbred Sprague–Dawley rats, previously shown to reach daily levels of alcohol intake of 4–6 g/kg under the intermittent access conditions used here,^37^ were given intermittent access to alcohol under a two‐bottle choice procedure as previously described.^37^, ^38^ During each session, rats had free access to two bottles containing either 10% alcohol or water for 24 h in their home cage. During the intermitting 24‐ or 48‐h alcohol‐free periods, rats had access to two bottles of water, resulting in an opportunity to drink alcohol three times a week. The location of the alcohol bottle was switched between sessions to avoid any side preference bias.

The bottles were weighed before and immediately after each two‐bottle choice session, and the volume consumed per session was computed as the weight difference between the start and the end of the sessions minus the spillage volume, accounted for the volume of fluid lost from bottles placed on an empty cage.^40^, ^41^

During the alcohol adulteration tests, alcohol solution was substituted with alcohol containing 0.1 g/L quinine for 24 h (alcohol + quinine vs. water).

During the saccharin choice test, saccharin 0.2% (v/v) was added to the water bottle for 24 h (alcohol vs. water + saccharin).

### 2.5 Tissue collection and RNA extraction

Rats were deeply anesthetized by 5% isoflurane inhalation and decapitated. Brains were harvested, frozen by immersion in −35/40°C isopentane for ~3 min, and stored at −80°C. Bilateral samples from the CeA and BLA were collected using a micro‐puncher (1 mm) from 300‐μm‐thick coronal sections obtained using a cryostat and stored at −80°C. RNAs were extracted using the Quick‐RNA™ Microprep kit (Zymo Research) following the manufacturer guidelines and quantified using the NanoDrop® ND‐1000 spectrophotometer (Thermo Fisher Scientific).

### 2.6 Quantitative polymerase chain reaction (qPCR)

RNA was reverse‐transcribed into cDNA with the RT^2^ First Strand Kit (Qiagen, UK) according to the manufacturer instructions. The following primers were used to assess the relative level of GAT‐3 mRNA: Slc6a11 Rn.10545 in comparison to that of cyclophilin A (Peptidylprolyl isomerase A) used as housekeeping gene (Qiagen, UK). Real‐time‐PCR was performed on the CFX96 Real‐Time PCR Detection System (Bio‐Rad, UK) using the RT^2^ SYBR Green Mastermix (Qiagen, UK) under the following conditions: reaction volume of 25 μl (1 μl cDNA, 24 μl PCR reaction mixture), 1 initial step at 95°C (10 min) followed by 40 temperature cycles (95°C for 15 s, 60°C for 60 s).

The relative mRNA level of the target gene was calculated using CFX Manager Software (Bio‐Rad, UK) and expressed as 2^‐ΔCT^ ^46^, ^47^ relative to cyclophilin A.

### 2.7 Data and statistical analysis

Data are presented as boxplots (median ± 25% [percentiles] and Min/Max as whiskers). The individual propensity to persist in drinking alcohol (as % of baseline) was calculated as the ratio between alcohol + quinine (Alcohol adulteration test) or alcohol (Saccharin choice test) intake (g/kg) over the average alcohol intake during the last three pre‐test baseline sessions.

Statistical analyses were performed with STATISTICA 10 software (StatSoft, Inc., Tulsa, OK, USA). Assumptions for parametric analysis, namely, normality of distribution, homogeneity of variance, and sphericity, were verified prior to each analysis with Shapiro–Wilk, Kolmogorov–Smirnov, and Cochran and Mauchly's tests, respectively. Where normality was substantially violated, data were square root transformed.

K‐means cluster analysis, carried out as previously described,^40^, ^41^, ^42^ on the persistence in alcohol drinking despite adulteration or access to saccharin, identified three specific subpopulations: AAR, AAI, and AAS.

Escalation of alcohol intake (g/kg) over repeated sessions of intermittent access was analyzed at the population level with repeated‐measures analysis of variance (ANOVA) with time (nine sessions) as the within‐subject factor. The magnitude of escalation across AAR and AAS rats was analyzed by ANOVA with time (first and third weeks) as the within‐subject factor and group (AAR vs. AAS) as the between‐subject factor.

Persistence in alcohol drinking (as % of baseline) in the adulteration and saccharin choice tests was analyzed with repeated‐measures ANOVA with test as within‐subject factor and group (AAR vs. AAS) as between‐subject factor. The effect of baclofen on alcohol and water intake (g/kg) on the whole population was subjected to repeated‐measures ANOVA with treatment (three doses) as within‐subject factor. The differential effect of baclofen on AAR and AAS rats on baseline alcohol and water intake (g/kg and ml/kg) or compulsive alcohol drinking (% of baseline) was analyzed using a similar ANOVA with group (AAR vs. AAS) or group (AAR vs. AAS) and treatment (two doses) as a between‐subject factor, respectively. Total alcohol intake (g/kg), body weight, and GAT‐3 mRNA levels were analyzed with a one‐way ANOVA with group (AAR vs. AAS) as between‐subject factor.

Dimensional analyses were performed with a Pearson correlation analysis with baseline alcohol intake (g/kg), adulterated alcohol intake (g/kg), adulterated alcohol intake (% baseline or square root of % baseline), and GAT‐3 mRNA levels as variables on the whole population, AAR, and AAS subpopulations.

For all analyses, upon confirmation of main effects, differences among individual means were further analyzed using Newman–Keuls post‐hoc test. Significance was set at *α* ≤ 0.05. Effect sizes are reported using partial eta‐squared value (*pη* ^2^).^42^

## 3 RESULTS

**FIGURE 2:**
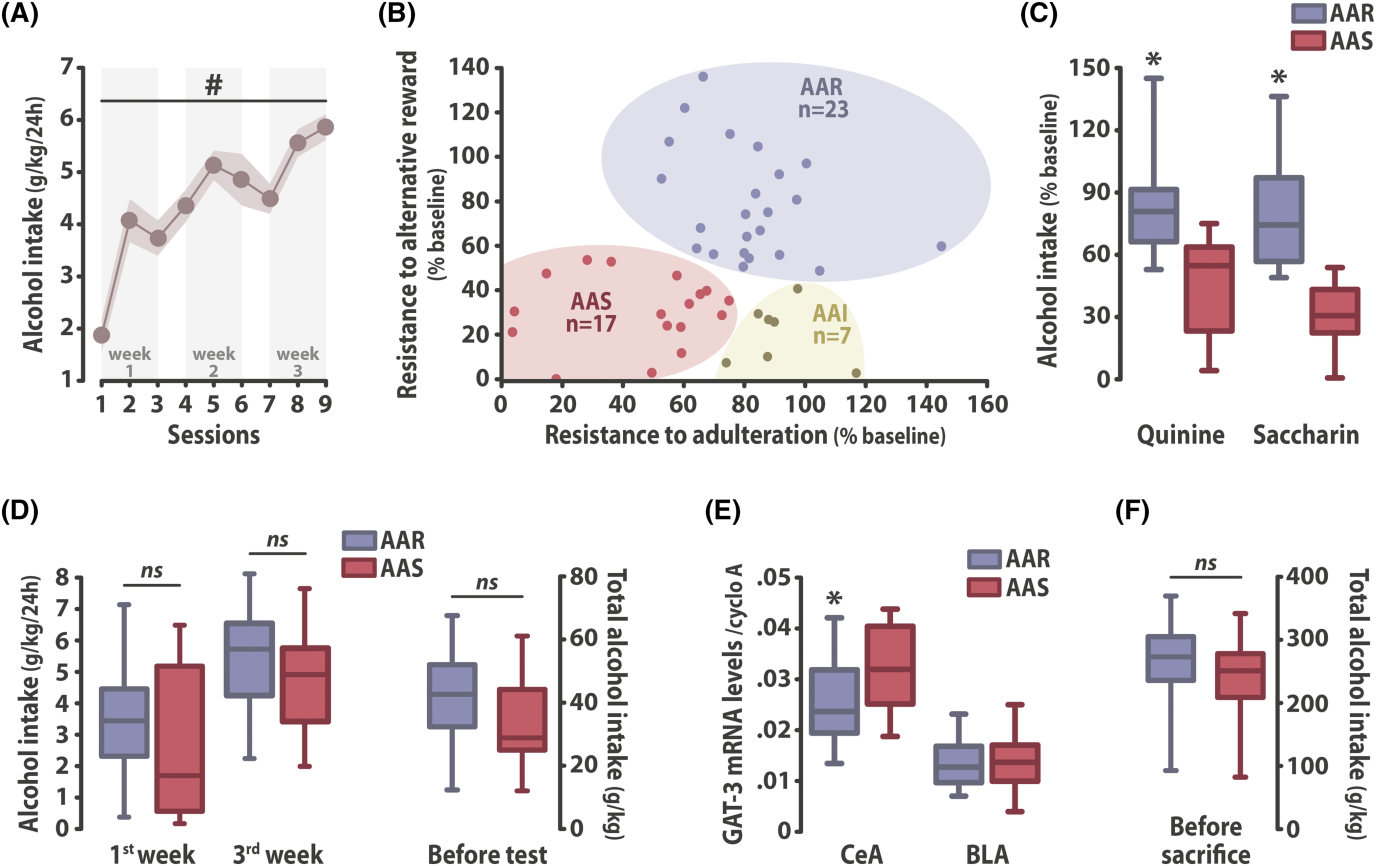
The vulnerability compulsively to drink alcohol is underlined by a low GAT‐3 mRNA level in the central amygdala. (A) Sprague–Dawley rats (*n* = 47) exposed to 10% alcohol in the two‐bottle choice procedure escalated their alcohol intake over the course of 3 weeks, eventually to reach ~5.8 g/kg/24 h after nine sessions. (B) A cluster analysis on both the persistence in alcohol drinking despite adulteration with quinine and access to a saccharin solution, identified three subpopulations, namely Adulteration‐Alternative reward Resistant (AAR, *n* = 23), Intermediate (AAI, *n* = 7), or Sensitive (AAS, *n* = 17) rats. (C) While AAS rats substantially decreased their alcohol intake when alcohol was adulterated or saccharin was offered as an alternative, the AAR subpopulation maintained high levels of alcohol intake in both tests. These differential behavioral manifestations were not attributable to differences between AAR and AAS rats in their escalation of alcohol intake over the previous 3 weeks (D, left panel) or in their overall alcohol exposure before the tests (D, right panel). (E) AAR rats showed lower GAT‐3 mRNA levels than the AAS rats in the CeA (*n* = 22 and *n* = 17, respectively) (left panel) but not in the BLA (*n* = 21 and *n* = 17, respectively) (right panel). The lower GAT‐3 mRNA levels shown by AAR rats in the CeA were not attributable to a differential alcohol exposure prior to sacrifice (F). # *p* < 0.01 main effect of session, * *p* < 0.05 different from AAS rats. BLA: Basolateral amygdala; CeA: Central amygdala; cyclo A: cyclophilin A; ns: no significant

Forty‐seven rats exposed to intermittent access to 10% alcohol progressively escalated their alcohol intake (main effect of session: *F*_8,368_ = 23.873, *p* < 0.001, *pη* ^2^ = 0.34), eventually to reach 5.8 g/kg/24 h after three weeks of exposure (Figure 2A). Then, the persistence of alcohol drinking after adulteration with quinine or when saccharin‐sweetened water was available as an alternative reward was tested in each individual (Figure 1).

Marked individual differences were observed in the persistence of alcohol drinking in each of the two tests that led to the characterization by cluster analysis of three subpopulations, namely Adulteration‐Alternative reward Resistant (AAR, n = 23), Intermediate (AAI, n = 7) and Sensitive (AAS, n = 17).

AAR and AAS were considered vulnerable and resilient to develop compulsive alcohol drinking, respectively (Figure 2B) since the former were resistant to both challenges which produced a significant decrease in alcohol intake in the latter (main effect of group: *F*_1,38_ = 95.322, *p* < 0.001, *pη* ^2^ = 0.71; test x group interaction: *F*_1,38_ = 1.346, *p* = 0.253) (Figure 2C). This differential vulnerability to develop compulsive alcohol drinking was not attributable to differences in the escalation of alcohol intake before these challenges. AAR rats did not differ from their AAS counterparts in their pattern of escalation (main effect of time: *F*_1,38_ = 63.932, *p* < 0.001, *pη* ^2^ = 0.63; time x group interaction: *F*_1,38_ < 1) (Figure 2D, left panel) and overall amount of alcohol intake (main effect of group: *F*_1,38_ = 2.6893, *p* = 0.109) (Figure 2D, right panel). AAR and AAS did not differ in their body weights either (main effect of group: *F*_1,38_ = 2.0772, *p* = 0.158, data not shown).

As predicted, AAR rats showed a lower level of GAT‐3 mRNA in the CeA than the AAS rats (main effect of group: *F*_1,37_ = 4.564, *p* = 0.039, *pη* ^2^ = 0.11) but not in the BLA (main effect of group: *F*_1,36_ < 1) (Figure 2E). The decreased GAT‐3 mRNA levels in the CeA shown by AAR rats were specifically related to their compulsive alcohol drinking and not due to a differential level of alcohol exposure prior to sacrifice (main effect of group: *F*_1,38_ = 2.139, *p* = 0.152) (Figure 2F).

**FIGURE 3:**
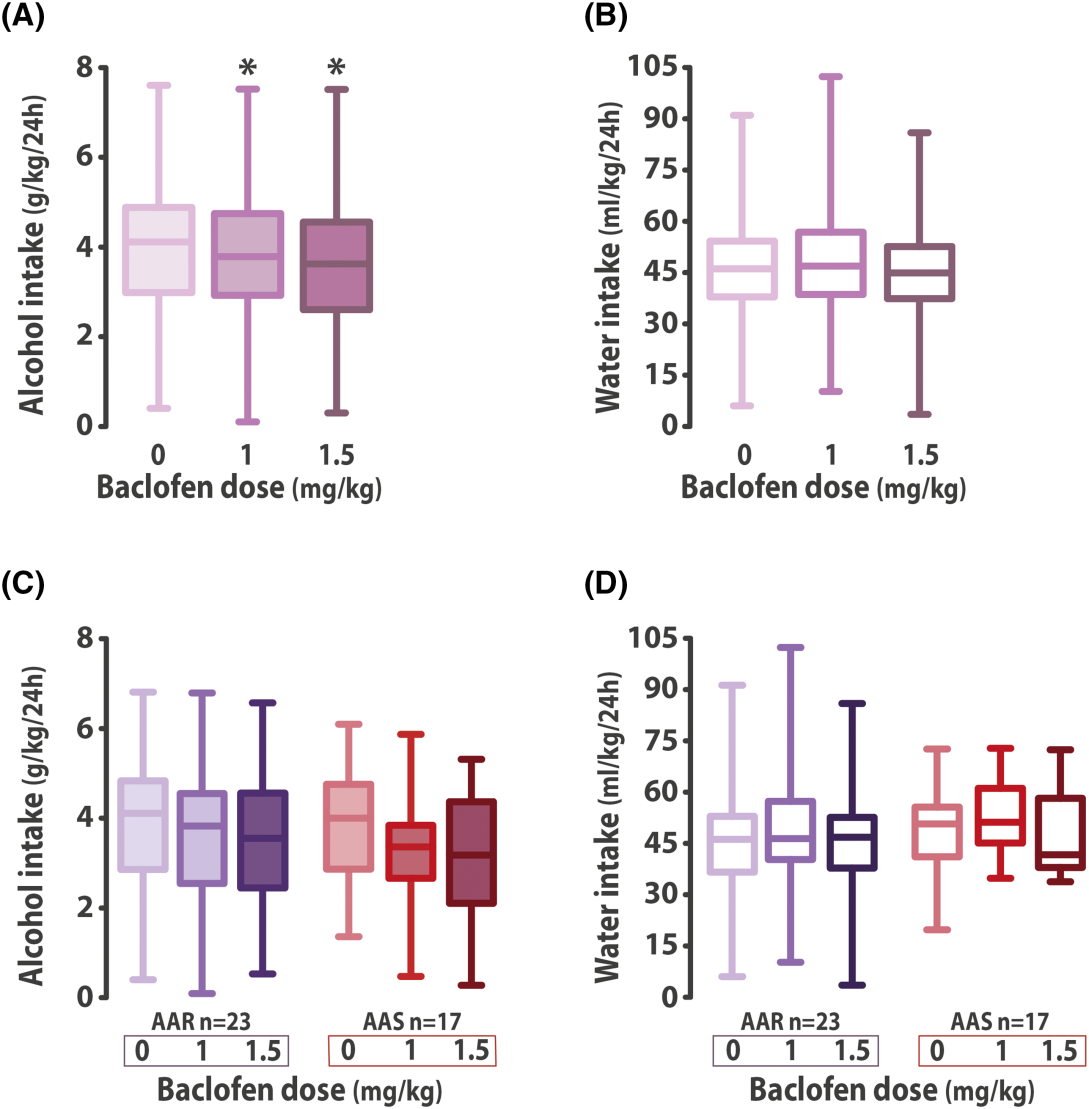
Baclofen reduces voluntary alcohol drinking in a heterogeneous rat population. Systemic administration of baclofen (1 and 1.5 mg/kg, intraperitoneal) reduced voluntary alcohol (A) but not water intake (B) in the two‐bottle choice procedure in a population of 47 rats. The decrease in voluntary alcohol intake after baclofen administration was not driven by either the AAR (*n* = 23) or the AAS rats (*n* = 17), since both groups displayed similar reduction in alcohol (C) while maintaining constant water intake (D) when treated with baclofen. * *p* < 0.05 compared with saline treatment (0 mg/kg group). AAR: Adulteration‐Alternative reward Resistant rats; AAS: Adulteration‐Alternative reward Sensitive rats

Baclofen induced, at the population level, a decrease in the voluntary intake of alcohol (main effect of treatment: *F*_2,92_ = 4.691, *p* = 0.011, *pη* ^2^ = 0.09) (Figure 3A) but not water (main effect of treatment: *F*_2,92_ = 1.515, *p* = 0.225) (Figure 3B).

**FIGURE 4:**
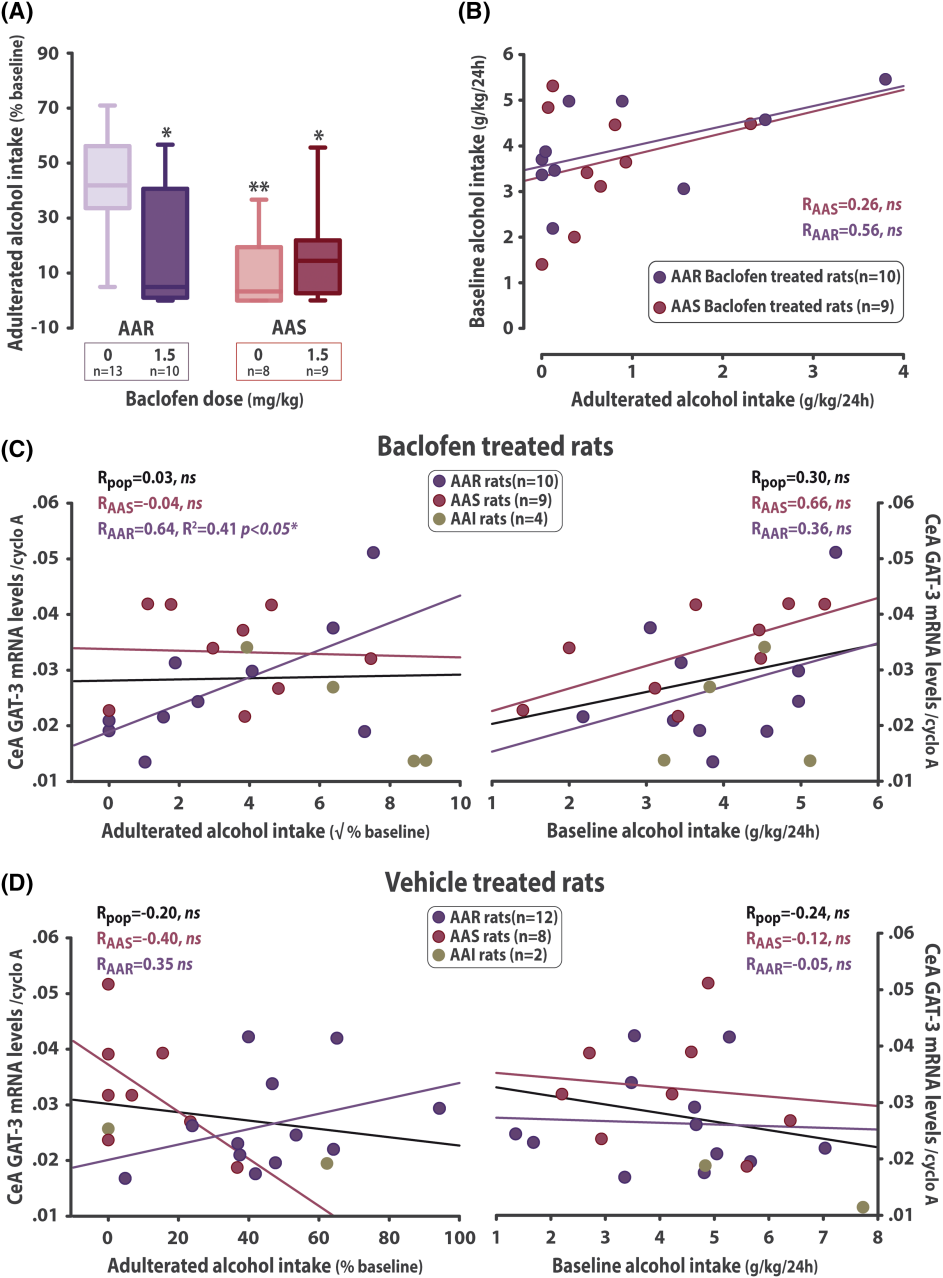
Baclofen selectively decreases persistent, compulsive alcohol drinking. (A) The compulsive phenotype of AAR rats was maintained after four additional months of intermittent exposure to alcohol as revealed by their persistence of drinking despite adulteration with quinine. Baclofen (1.5 mg/kg, intraperitoneal) selectively decreased compulsive drinking in AAR rats while having no influence on the alcohol drinking shown by the AAS subpopulation to be substantially decreased by adulteration. (B) The selective effect baclofen was shown to exert on persistent drinking in AAR rats was absolutely independent of, and unrelated to, that baclofen had on drinking under baseline conditions. In ARR rats only, GAT‐3 mRNA levels in the CeA predicted the response of compulsive (C, left panel) but not baseline drinking to baclofen (C, right panel). This observation was specific to AAR rats treated with baclofen since no correlations were observed between GAT‐3 mRNA levels and compulsive (D, left panel) or baseline alcohol drinking (D, right panel) in rats that had received vehicle. * *p* < 0.05, ** *p* < 0.01 compared with the saline treated (0 mg/kg) AAR group. AAR: Adulteration‐Alternative reward Resistant rats; AAI: Adulteration‐Alternative reward Intermediate rats; AAS: Adulteration‐Alternative reward Sensitive rats; CeA: central amygdala; cyclo A: cyclophilin A; pop: whole population; ns: no significant; √: square root

The specific reduction in baseline alcohol intake by systemic administration of baclofen was of similar magnitude between AAR and AAS rats (main effect of treatment: *F*_2,76_ = 7.655, *p* < 0.001, *pη* ^2^ = 0.17; treatment x group interaction: *F*_2,76_ < 1) (Figure 3C), having no effect on water intake (main effect of treatment: *F*_2,76_ = 2.370, *p* = 0.100; treatment x group interaction: *F*_2,76_ < 1) (Figure 3D). In marked contrast, baclofen selectively reduced compulsive, quinine‐resistant alcohol drinking shown by AAR rats when both groups where challenged with another quinine adulteration test four months later (main effect of group: *F*_1,36_ = 7.07, *p* = 0.012, *pη* ^2^ = 0.16; group x treatment interaction: *F*_1,36_ = 5.441, *p* = 0.025, *pη* ^2^ = 0.13) (Figure 4A). The selective sensitivity displayed by AAR rats to baclofen when they expressed persistent, compulsive drinking was not correlated with that they showed for their unchallenged drinking at baseline (*R* = 0.5617, *p* = 0.091); no correlation was observed for AAS rats either (*R* = 0.2583, *p* = 0.502) (Figure 4B). However, in ARR rats, GAT‐3 mRNA levels in the CeA predicted the response of compulsive (*R* = 0.64, *R* ^2^ = 0.41, *p* < 0.05), but not baseline drinking (*R* = 0.36, *p* = 0.310), to baclofen (Figure 4C). This relationship, which was not observed in vehicle‐treated AAR rats (*R* = 0.35, *p* = 0.259) (Figure 4D), was specific to the AAR rats treated with baclofen as no correlation was observed for vehicle‐ or baclofen‐treated rats between GAT‐3 mRNA levels in the CeA and compulsive (AAS‐baclofen: *R* = −0.04, *p* = 0.916; AAS‐vehicle: *R* = −0.40, *p* = 0.318; whole population‐baclofen: R = 0.03, *p* = 0.893; whole population‐vehicle: *R* = −0.20, *p* = 0.381), or baseline alcohol drinking (AAS‐baclofen: *R* = 0.66, *p* = 0.056; AAS‐vehicle: *R* = −0.12, *p* = 0.781; whole population‐baclofen: *R* = 0.30, *p* = 0.160; whole population‐vehicle: *R* = −0.24, *p* = 0.272) (Figure 4C, D).

## 4 DISCUSSION

The results of this study reveal that following intermittent two‐bottle choice exposure to 10% alcohol,^37^, ^38^ a large population of Sprague–Dawley rats escalated their alcohol intake over three weeks, reaching levels of alcohol consumption similar to that described across different strains in previous studies that used 20% alcohol,^37^ including the original demonstration by Wise.^38^ Thus, even though it requires ingestion of twice as much liquid when exposed to 10% instead of 20% alcohol, Sprague–Dawley rats readily drank enough alcohol to reach levels of intake higher than those previously reported to result in blood alcohol concentrations of 30 mg%.^37^ However, only a subset of vulnerable individuals that were characterized by low GAT‐3 mRNA levels in the CeA went on to persistently drink alcohol when adulterated with quinine^29^, ^31^, ^32^, ^48^ or when an alternative, highly preferred sweet reward was available.^17^, ^33^, ^49^

These resistant rats displayed behaviors that have been suggested to be the operationalization in rodents of two features that characterize compulsive alcohol drinking, namely, persistence of drinking despite adverse consequences and decreased interest for reinforcers other than the drug itself.^29^, ^32^, ^33^

In line with previous studies,^32^, ^34^, ^41^ the development of compulsive drinking behavior in vulnerable animals was shown not to be predicted by the individual tendency to drink high levels of alcohol, thereby confirming that the development of this behavioral feature of alcohol addiction is not simply related to the magnitude of pharmacological exposure to alcohol.

This dissociation, which challenges a drug‐centered view of drug addiction and further emphasizes the importance of factors of vulnerability,^50^, ^51^ is not restricted to alcohol. The propensity to switch from controlled to cocaine addiction‐like behaviors in rats is also independent of the level of cocaine intoxication,^18^, ^52^ while a history of escalated cocaine intake does not facilitate the development of compulsive cocaine self‐administration^53^ or compulsive cocaine seeking.^52^

These inter‐individual differences in the propensity to drink alcohol despite its adulteration or the availability of a sweet ingestive reward that emerged after three  weeks of intermittent access to alcohol under a two‐bottle choice procedure persisted for at least four months. This supports the construct validity of the experimental design used here and suggests that adaptations at the psychological and neural systems level that result in the emergence of compulsive alcohol drinking (present study) and seeking^40^, ^41^ persist over time regardless of further intoxication.

Previous studies^30^, ^31^, ^39^, ^43^ have suggested that more prolonged exposure to alcohol (three to six months^43^) is necessary for the emergence of a quinine‐resistant phenotype in rats, whereas this characteristic appeared more rapidly in the present study. However, even though the dose of quinine used here is similar to that used in the previous studies, several important methodological differences preclude any direct comparison. In the present study, quinine resistance was measured in a home cage‐based non‐operant procedure, as in the seminal studies of Wolffgramm and colleagues.^31^, ^39^ However, in their intermittent conditions, alcohol was available only once a week as opposed to three times a week in the present study. On the other hand, when rats had been trained to drink alcohol under similar intermittent access conditions to those used here, the effect of quinine was not assessed on drinking under free access conditions but on instrumental performance under a progressive ratio of alcohol reinforcement.^43^ Additionally, in previous studies, the resistance to quinine adulteration was measured at the population level and did not factor in the individual vulnerability that clearly emerged in the design of the present study.

This individual vulnerability to develop compulsive alcohol drinking behavior was shown here to be associated with adaptations in the GABAergic system in the CeA. As compared to AAS rats, AAR rats showed reduced mRNA levels of the GABA transporter GAT‐3 in the CeA but not the BLA. This difference could not be attributed to differential exposure to alcohol or to differences in the escalation of alcohol intake, since it was similar between AAR and AAS; therefore, it either predates the exposure to alcohol or emerges as vulnerability‐specific alcohol‐induced adaptation and can therefore be seen as a biological marker of the vulnerability to develop compulsive alcohol drinking.

These findings are consistent with the previous demonstration of altered expression of several genes involved in GABAergic transmission, including lower GAT‐3 mRNA levels, in the amygdala of rats characterized as choosing alcohol over an alternative reward, as well as resisting punishment and quinine adulteration in an instrumental setting.^17^ Together, these observations suggest that alterations in GABAergic mechanisms in the CeA underlie the compulsive nature of both consummatory and preparatory responses for alcohol, which are otherwise psychologically and neurobiologically dissociable.^54^, ^55^ Therefore, beyond compulsivity, an altered GABAergic system in the CeA may also be a biological marker of the vulnerability to develop alcohol addiction. This suggestion is further supported by the presence of similar alterations in GAT‐3 in humans with AUD.^17^

GABA transporters are involved in the clearance of GABA from the synaptic cleft and therefore play an important role in regulating the extracellular concentration of GABA.^20^, ^49^ Indeed, downregulation of GAT‐3 in rats showing addiction‐like behaviors results in an increased GABAergic tone in the CeA, presumably as the result of decreased GABA clearance by these GABA transporters in the synaptic cleft.^17^

Pharmacological activation of GABA_B_ receptors by the agonist baclofen reduces the extracellular release of GABA^23^, ^24^ and results in a decrease in alcohol intake at the population level, as shown in this and in previous studies.^6^ Furthermore, baclofen selectively decreased the compulsive drinking of alcohol by AAR rats when challenged by adulteration with quinine, an effect that was associated with the GAT‐3 mRNA levels in the CeA in that the higher the GAT‐3 mRNA levels, the greater the resistance to quinine in baclofen‐treated but not vehicle‐treated compulsive rats.

This effect was not attributable to a non‐specific effect of baclofen on the aversive properties of quinine because the degree of suppression of drinking following quinine adulteration was not affected by this treatment in AAS rats. This effect was also independent of the influence of baclofen on alcohol drinking by AAR rats at baseline in a two‐bottle choice session, as there was no correlation between the suppressant effect on drinking between the two conditions.

Together, these data suggest that voluntary alcohol drinking and persistent alcohol drinking in the face of negative consequences depend on distinct GABAergic mechanisms. This observation is in agreement with the finding that rats with three addiction‐like behaviors in a multisymptomatic preclinical model^19^ of AUD were specifically sensitive to the motivation‐ and reinstatement‐suppressing effects of baclofen.^51^

Taken together, these results suggest that the decrease in compulsive seeking^51^ and drinking (present study) behaviors shown when individuals are treated with baclofen may be mediated by a normalization of impaired GABA clearance resulting from the decreased GAT‐3 levels. It is important to note, however, that baclofen was administered systemically, thereby precluding any conclusion with regards to its neural locus of action. Further investigations will be necessary to establish whether restoration of GABA levels, specifically and exclusively within the CeA, is necessary and sufficient to decrease compulsive alcohol seeking or drinking.

The psychological consequences of altered GABAergic physiology in the CeA are not fully understood. However, a downregulation of GAT‐3 mRNA levels in the CeA of rats has been associated with increased anxiety,^17^ in line with the long‐established role of the CeA and its GABAergic system in the expression of anxiety‐related disorders.^56^, ^57^ Considering that anxiety and associated alcohol drinking as a self‐medication strategy are important factors of vulnerability in the development of AUD,^48^, ^50^, ^58^, ^59^ an altered CeA GABAergic system may represent an endophenotype of this individual vulnerability.

The results of the present study provide new evidence that compulsive alcohol drinking is associated with decreased GAT‐3 mRNA levels and is selectively suppressed by treatment with baclofen.

## AUTHORS CONTRIBUTION

LMP and DB designed the experiments. LMP, MF, MP, and PJC carried out the behavioral experiment. LMP and ABR performed the molecular procedures. LMP and MF performed the data analysis. LMP and DB wrote the manuscript. BJE gave intellectual input and contributed to the redaction of the manuscript. All authors have critically reviewed content and approved final version submitted for publication.
